# Crystal structure of (1*Z*,2*Z*)-*N*^1^,*N*^2^-diisobutyl-1,2-di­phenyl­ethane-1,2-di­imine

**DOI:** 10.1107/S2056989026002227

**Published:** 2026-03-05

**Authors:** Naser E. Eltayeb, Jamal Lasri, Yaseen A. Almehmadi, Tuncer Hökelek, Aidan P. McKay

**Affiliations:** a Department of Chemistry Rabigh College of Science and Arts King Abdulaziz University,Jeddah 21589 Saudi Arabia; b Department of Chemistry, Faculty of Pure and Applied Sciences, International University of Africa, Khartoum 2469, Sudan; c King Fahd Medical Research Center King Abdulaziz University,Jeddah 21589 Saudi Arabia; d Department of Physics, Hacettepe University, 06800 Beytepe, Ankara, Türkiye; e EaStCHEM School of Chemistry, University of St Andrews, Fife KY16 9ST, United Kingdom; Katholieke Universiteit Leuven, Belgium

**Keywords:** (1*Z*,2*Z*)-*N*^1^,*N*^2^-diisobutyl-1,2-di­phenyl­ethane-1,2-di­imine, crystal structure, C—H⋯π inter­action

## Abstract

The asymmetric unit of the title compound, C_22_H_28_N_2_, contains 1,2-di­phenyl­ethane-1,2-di­imine and diisobutyl groups, with the dihedral angle between the phenyl rings being 89.23 (5)°. In the crystal, the mol­ecules are elongated along the *c*-axis direction and stacked along the *b*-axis direction. Neither intra- or inter­molecular hydrogen bondings nor aromatic π–π stacking inter­actions are observed. The weak C—H⋯π(ring) inter­actions may help in the consolidation of the packing.

## Abstract 2

In the title compound, the dihedral angle between the phenyl rings is 89.23 (5)°. In the crystal, the mol­ecules are elongated along the *c*-axis direction and stacked along the *b*-axis direction. Neither intra- or inter­molecular hydrogen bondings nor π–π inter­actiones are observed. The weak C—H⋯π(ring) inter­actions may help in the consolidation of the packing.

## 1. Chemical context

Benzil [*i.e.* Bz_2_, known as 1,2-di­phenyl­ethane-1,2-dione, (C_6_H_5_CO)_2_, generally abbreviated as (PhCO)_2_] is a common building block in synthetic organic chemistry, which is also known to be a potent inhibitor of mammalian carboxyl­esterase enzymes (Wadkins *et al.*, 2005 ▸). The condensation reaction of *o*-amino­phenol, 2-amino­ethanol and their related compounds, containing S or N atoms instead of O, with α-diketones has been the topic of various research publications (Schminpeter & Winmaier, 1975 ▸; Singh *et al.*, 1990 ▸; Marjani *et al.*, 2007 ▸). For instance, the treatment of benzil with 4-amino­anti­pyridine or *o*-amino­phenol affords a Schiff base adduct or an unexpected oxazine derivative, respectively. Additionally, both compounds exhibit promising anti­cancer activity against HepG2 and MCF-7 cell lines (Lasri *et al.*, 2023 ▸). Benzil-based simple Schiff base probes were developed for selective colorimetric Cu^2+^ ions detection (Gogoi *et al.*, 2025 ▸). As part of our work in this area, we now report the mol­ecular and crystal structures of the title compound (I).
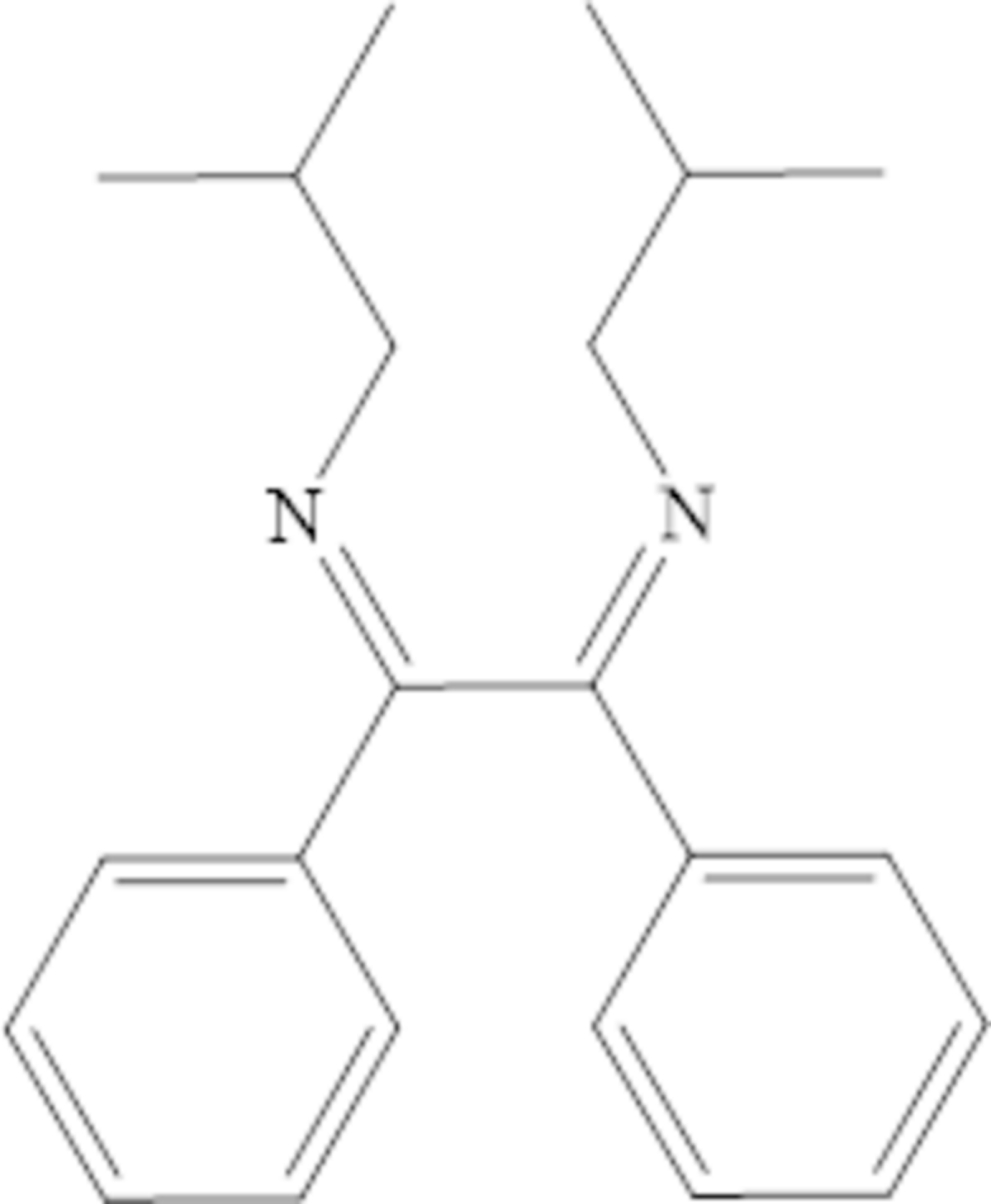


## 2. Structural commentary

**Figure 1:**
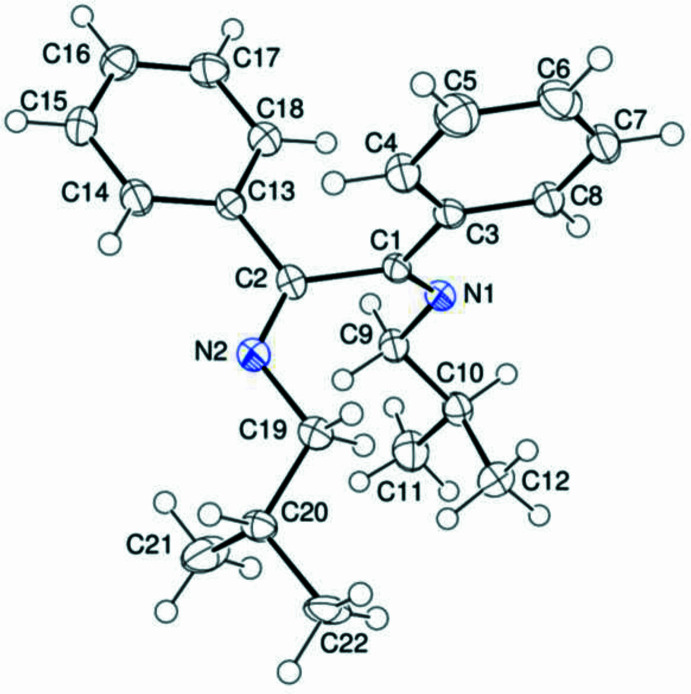
The title mol­ecule with atom-numbering scheme and 50% probability ellipsoids. Only the major component is shown for the disordered isopropyl group C20–C22.

The asymmetric unit of (I) contains 1,2-di­phenyl­ethane-1,2-di­imine and diisobutyl groups (Fig. 1 ▸). One of the isopropyl groups (C20–C22) is disordered over two sets of sites with occupancies of 0.759 (7)/0.241 (7). In the dibenzyl moiety, there are no unusual bond distances or inter-bond angles. In the 1,2-di­imine and diisobutyl moieties, the bond angles C1—N1—C9 [120.48 (11)°] and C2—N2—C19 [118.82 (12)°], and N1—C9—C10 [110.35 (11)°], N2—C19—C20 [112.00 (13)°] and N2—C19—C20*A* [114.3 (3)°] are significantly different. The same is true for the torsion angles C9—N1—C1—C2 [−2.99 (19)°] and C19—N2—C2—C1 [−0.8 (2)°], and C9—N1—C1—C3 [178.88 (11)°] and C19—N2—C2—C13 [−179.18 (12)°]. The two almost planar phenyl rings, *A* (C3–C8) and *B* (C13–C18), are perpendicularly oriented at a dihedral angle of *A*/*B* = 89.23 (5)°. On the other hand, atoms C1, N1, C9 and C2, N2, C19 are 0.0354 (13), 0.1065 (12), 0.1146 (14) Å and 0.0278 (14), −0.2595 (12), −0.2097 (15) Å, respectively, away from the corresponding best least-squares ring planes.

## 3. Supra­molecular features

**Figure 2:**
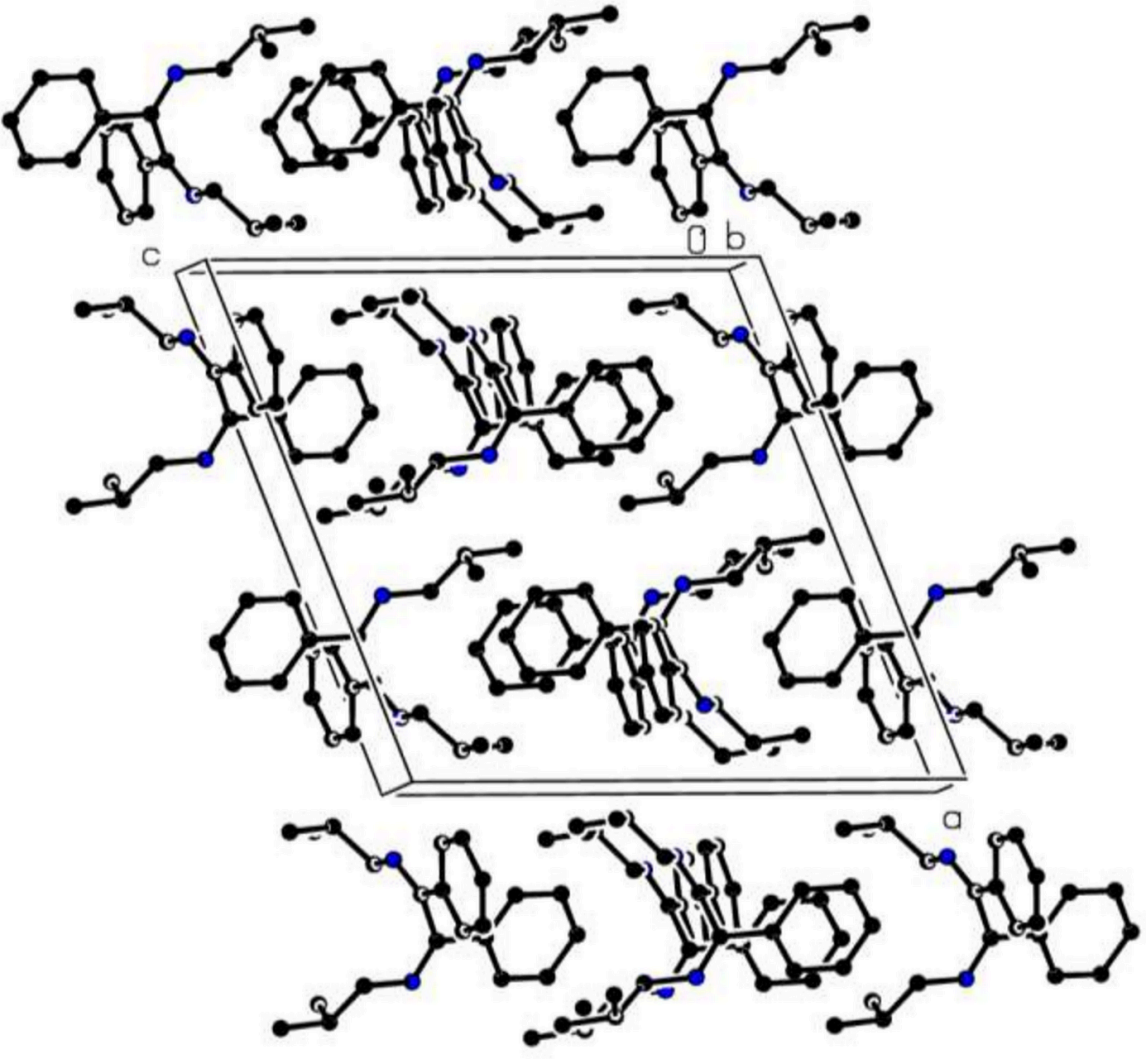
A partial packing diagram viewed down the *b*-axis direction. Hydrogen atoms have been omitted for clarity.

In the crystal, the mol­ecules are elongated along the *c*-axis direction and stacked along the *b*-axis direction (Fig. 2 ▸). Neither intra- or inter­molecular hydrogen bondings nor π–π inter­actiones are observed. Three weak C—H⋯π(ring) inter­actions (Table 1 ▸) may help to consolidate of the packing.

## 4. Database survey

A survey of the Cambridge Structural Database (CSD, July 2025 update; Groom *et al.*, 2016 ▸) revealed 12 structures similar to the title compound (1*Z*,2*Z*)-*N*^1^,*N*^2′^-diisobutyl-1,2-di­phenyl­ethane-1,2-di­imine, **I**. These most relevant structures include: compound **II** [CSD refcode ZOWKAZ; (1S^*^, 2R^*^)-*N,N,N′,N′*-tetra­benzyl-1,2-di­phenyl­ethane-1,2-di­amine toluene solvate; Hermant *et al.*, 2014 ▸], compound **III** {YOWFUM; *N,N′*-(1,2-diphenyl-1,2-ethane­diyl­idene)bis­[4-(2-thien­yl)aniline] di­chloro­methane solvate: Powell *et al.*, 2009 ▸}, compound **IV** (XOFNAJ; 2,2′-{1,2-bis­[(3,3-di­methyl­butan-2-yl)imino] ethane-1,2-di­yl}diphenol; Seo *et al.*, 2014 ▸), compound **V** {UVELEP; 4,4′-[(1,2-di­phenyl­ethane-1,2-di­yl) bis­(aza­nylylidenemethanylyl­idene)]bis­(*N,N′*-di­methyl­aniline); Shiju *et al.*, 2021 ▸}, compound **VI** {TAHHUI; 4,4′-[(1,2-dipheyl­ethane-1,2- diyl­idene)di­aza­nylyl­idene]di­cyclo­hexa­nol; Greb *et al.*, 2016 ▸}, compound **VII** {SATBAT; *N,N′*-[(*R*,*R*)-1,2-di­phenyl­ethane-1,2-di­yl]bis­[1-(9-anthr­yl)methanimine]; Bar­wiolek *et al.*, 2017 ▸}, compound **VIII** [RIRHAC; di­chloro-(1,2-bis­(cyclo­hexyl­imino)-1,2-di­phenyl­ethane-*N,N′*-iron(II) meth­anol solvate; Allan *et al.*, 2007 ▸], compound **IX** (BZYPEN; *N*,*N′*-di­benzyl­idene-1,2-di­phenyl­ethyl­enedi­amine; Prelesnik & Nowacki, 1975 ▸), compound **X** [ILISOL; *N,N′*-bis­(salicyl­idene)-1,2-(1*S*,2*S*)-(–)-diphenyl-1,2-ethanedi­amine; Korendovych & Rybak-Akimova, 2003 ▸], compound **XI** [IMUWAR; 1,2-bis­(4-meth­oxy­phen­yl)-*N^1^,N^2^*-di­phenyl­ethane-1,2-di­imine; Schuh *et al.*, 2021 ▸], compound **XII** [IXUDIR; 1,2-diphenyl-*N^1^,N^1^,N^2^,N^2^*-tetra­kis­(propan-2-yl)ethene-1,2-di­amine; Sobczak *et al.*, 2021 ▸] and compound **XIII** [KIFDEK; *N,N′*-1,2-di­phenyl­ethane-1,2-diyl­idene)bis­(4-meth­oxy­aniline); Kubota *et al.*, 2013 ▸].

The dihedral angles between the planes of the phenyl rings of the core benzil fragment vary over the range 0.0 to 89.23 (5)° due to the differing packings resulting from the varied sizes and shapes of substituents (Table 2 ▸).

## 5. Synthesis and crystallization

To a solution of benzil (200.0 mg, 0.951 mmol) in EtOH (50 ml), iso­butyl­amine (139.1 mg, 1.902 mmol) was added, then the mixture was refluxed for 6 h. The precipitate formed was filtered off and the filtrate was evaporated *in vacuo* to give the desired (1*Z*,2*Z*)-N^1^,N^2^-diisobutyl-1,2-di­phenyl­ethane-1,2- di­imine. Colourless crystals suitable for X-ray analysis were obtained by slow evaporation of an ethanol solution. Yield: 89%. FT-IR (cm^−1^): 1667 (C=N). Analysis calculated for C_22_H_28_N_2_: C, 82.45; H, 8.81; N, 8.74. Found: C, 82.49; H, 8.79; N, 8.76.

## 6. Refinement

Crystal data, data collection and structure refinement details are summarized in Table 3 ▸. The C-bond hydrogen-atom positions were calculated geometrically at distances of 0.95 Å (for aromatic CH), 1.00 Å (for methine CH), 0.99 Å (for methyl­ene CH) and 0.98 Å (for methyl CH) and refined using a riding model by applying the constraints of *U*_iso_(H) = *k* × *U*_eq_(C), where *k* = 1.5 for methyl hydrogen atoms and k = 1.2 for the other hydrogen atoms. Atoms C20, C21, C22, H19*A*, H19*B*, H20, H21*A*, H21*B*, H21*C*, H22*A*, H22*B*, H22*C* and C20*A*, C21*A*, C22*A*, H19*C*, H19*D*, H20*A*, H21*D*, H21*E*, H21*F*, H22*D*, H22*E*, H22*F* are disordered over two positions, and they were refined with the occupancy ratio of 0.759 (7)/0.241 (7).

## Supplementary Material

Crystal structure: contains datablock(s) I. DOI: 10.1107/S2056989026002227/vm2325sup1.cif

Structure factors: contains datablock(s) I. DOI: 10.1107/S2056989026002227/vm2325Isup2.hkl

Supporting information file. DOI: 10.1107/S2056989026002227/vm2325Isup3.cml

CCDC reference: 2534247

Additional supporting information:  crystallographic information; 3D view; checkCIF report

## Figures and Tables

**Table 1:** Hydrogen-bond geometry (Å, °) *Cg*1 and *Cg*2 are the centroids of phenyl rings C3–C8 and C13–C18, respectively.

*D*—H⋯*A*	*D*—H	H⋯*A*	*D*⋯*A*	*D*—H⋯*A*
C6—H6⋯*Cg*2^i^	0.95	3.23	4.044 (5)	144
C16—H16⋯*Cg*1^ii^	0.95	2.95	3.76 (7)	144
C21—H21*C*⋯*Cg*2^iii^	0.98	3.00	3.72 (5)	131

Symmetry codes: (i) 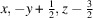; (ii) 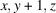; (iii) 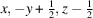.

**Table 2:** Comparison of the dihedral angle α (°) between the phenyl rings in some similar structures

Compound	α	Refcode
**I**	89.23 (5)	–
**II**	0.00	ZOWKAZ
**III**	84.27	YOWFUM
**IV**	87.64	XOFNAJ
**V**	0.00	UVELEP
**VI**	82.75	TAHHUI
**VII**	48.15	SATBAT
**VIII**	61.24	RIRHAC
**IX**	47.28	BZYPEN
***X***	29.60	ILISOL
**XI**	79.72	IMUWAR
**XII**	37.66	IXUDIR
**XIII**	79.97	KIFDEK

**Table 3:** Experimental details

Crystal data	
Chemical formula	C_22_H_28_N_2_
*M*_r_	320.46
Crystal system, space group	Monoclinic, *P*2_1_/*c*
Temperature (K)	100
*a*, *b*, *c* (Å)	14.9744 (4), 9.09166 (17), 14.8245 (4)
β (°)	111.189 (3)
*V* (Å^3^)	1881.80 (8)
*Z*	4
Radiation type	Mo *K*α
μ (mm^−1^)	0.07
Crystal size (mm)	0.12 × 0.09 × 0.05

Data collection	
Diffractometer	Rigaku XtaLAB P200K
Absorption correction	Multi-scan (*CrysAlis PRO*; Rigaku OD, 2024 ▸)
*T*_min_, *T*_max_	0.729, 1.000
No. of measured, independent and observed [*I* > 2σ(*I*)] reflections	40015, 4483, 3689
*R*_int_	0.039
(sin θ/λ)_max_ (Å^−1^)	0.684

Refinement	
*R*[*F*^2^ > 2σ(*F*^2^)], *wR*(*F*^2^), *S*	0.052, 0.135, 1.05
No. of reflections	4483
No. of parameters	251
No. of restraints	27
H-atom treatment	H-atom parameters constrained
Δρ_max_, Δρ_min_ (e Å^−3^)	0.46, −0.22

Computer programs: *CrysAlis PRO* (Rigaku OD, 2024 ▸), *SHELXT2018/2* (Sheldrick, 2015*a* ▸), *SHELXL2019/3* (Sheldrick, 2015*b* ▸) and *OLEX2* (Dolomanov *et al.*, 2009 ▸).

## supplementary crystallographic information

### (1Z,2Z)-N1,N2-Diisobutyl-1,2-diphenylethane-1,2-diimine Crystal data

**Table:**

C_22_H_28_N_2_	*F*(000) = 696
*M**_r_* = 320.46	*D*_x_ = 1.131 Mg m^−^^3^
Monoclinic, *P*2_1_/*c*	Mo *K*α radiation, λ = 0.71073 Å
*a* = 14.9744 (4) Å	Cell parameters from 15611 reflections
*b* = 9.09166 (17) Å	θ = 2.7–29.1°
*c* = 14.8245 (4) Å	µ = 0.07 mm^−^^1^
β = 111.189 (3)°	*T* = 100 K
*V* = 1881.80 (8) Å^3^	Prism, colourless
*Z* = 4	0.12 × 0.09 × 0.05 mm

### (1Z,2Z)-N1,N2-Diisobutyl-1,2-diphenylethane-1,2-diimine Data collection

**Table:**

Rigaku XtaLAB P200K diffractometer	4483 independent reflections
Radiation source: Rotating Anode, Rigaku FR-X	3689 reflections with *I* > 2σ(*I*)
Rigaku Osmic Confocal Optical System monochromator	*R*_int_ = 0.039
Detector resolution: 5.8140 pixels mm^-1^	θ_max_ = 29.1°, θ_min_ = 2.7°
shutterless scans	*h* = −19→19
Absorption correction: multi-scan (CrysAlisPro; Rigaku OD, 2024)	*k* = −11→10
*T*_min_ = 0.729, *T*_max_ = 1.000	*l* = −20→19
40015 measured reflections	

### (1Z,2Z)-N1,N2-Diisobutyl-1,2-diphenylethane-1,2-diimine Refinement

**Table:**

Refinement on *F*^2^	Primary atom site location: dual
Least-squares matrix: full	Hydrogen site location: inferred from neighbouring sites
*R*[*F*^2^ > 2σ(*F*^2^)] = 0.052	H-atom parameters constrained
*wR*(*F*^2^) = 0.135	*w* = 1/[σ^2^(*F*_o_^2^) + (0.0567*P*)^2^ + 0.9225*P*] where *P* = (*F*_o_^2^ + 2*F*_c_^2^)/3
*S* = 1.05	(Δ/σ)_max_ < 0.001
4483 reflections	Δρ_max_ = 0.46 e Å^−^^3^
251 parameters	Δρ_min_ = −0.22 e Å^−^^3^
27 restraints	

### (1Z,2Z)-N1,N2-Diisobutyl-1,2-diphenylethane-1,2-diimine Special details

**Table:**

Geometry. All esds (except the esd in the dihedral angle between two l.s. planes) are estimated using the full covariance matrix. The cell esds are taken into account individually in the estimation of esds in distances, angles and torsion angles; correlations between esds in cell parameters are only used when they are defined by crystal symmetry. An approximate (isotropic) treatment of cell esds is used for estimating esds involving l.s. planes.
Refinement. One terminal iso-propyl group was disordered and modelled over two sites with geometric restraints on minor component and some thermal restraints.

### (1Z,2Z)-N1,N2-Diisobutyl-1,2-diphenylethane-1,2-diimine Fractional atomic coordinates and isotropic or equivalent isotropic displacement parameters (Å^2^)

**Table:**

	*x*	*y*	*z*	*U*_iso_*/*U*_eq_	Occ. (<1)
N1	0.37234 (8)	0.86706 (13)	0.61972 (8)	0.0232 (3)	
N2	0.14025 (8)	0.81812 (13)	0.56906 (9)	0.0250 (3)	
C1	0.29496 (9)	0.85203 (14)	0.54690 (10)	0.0208 (3)	
C2	0.20808 (9)	0.76482 (15)	0.54711 (10)	0.0218 (3)	
C3	0.28760 (9)	0.91967 (14)	0.45281 (10)	0.0220 (3)	
C4	0.20704 (10)	0.90086 (16)	0.36928 (11)	0.0279 (3)	
H4	0.154265	0.845326	0.371846	0.034*	
C5	0.20313 (12)	0.96279 (18)	0.28193 (12)	0.0353 (4)	
H5	0.147825	0.949203	0.225345	0.042*	
C6	0.27952 (12)	1.04399 (17)	0.27735 (12)	0.0353 (4)	
H6	0.276944	1.085687	0.217703	0.042*	
C7	0.35985 (12)	1.06419 (16)	0.36023 (12)	0.0324 (3)	
H7	0.412320	1.120084	0.357258	0.039*	
C8	0.36407 (10)	1.00328 (15)	0.44748 (11)	0.0263 (3)	
H8	0.419212	1.018405	0.503993	0.032*	
C9	0.38136 (10)	0.80456 (15)	0.71334 (10)	0.0250 (3)	
H9A	0.407259	0.703423	0.718588	0.030*	
H9B	0.317420	0.799228	0.718680	0.030*	
C10	0.44799 (10)	0.89913 (16)	0.79551 (10)	0.0261 (3)	
H10	0.511786	0.904326	0.788291	0.031*	
C11	0.46138 (12)	0.82634 (19)	0.89208 (11)	0.0368 (4)	
H11A	0.398785	0.812680	0.897948	0.055*	
H11B	0.501575	0.889013	0.944960	0.055*	
H11C	0.492379	0.730505	0.895464	0.055*	
C12	0.40984 (11)	1.05480 (17)	0.79215 (12)	0.0322 (3)	
H12A	0.399703	1.098330	0.728731	0.048*	
H12B	0.456254	1.114151	0.842774	0.048*	
H12C	0.348954	1.052269	0.802784	0.048*	
C13	0.20366 (10)	0.60699 (15)	0.51653 (10)	0.0227 (3)	
C14	0.11641 (10)	0.53135 (16)	0.48865 (11)	0.0281 (3)	
H14	0.060975	0.579783	0.490742	0.034*	
C15	0.11025 (11)	0.38670 (17)	0.45808 (12)	0.0335 (4)	
H15	0.050625	0.336522	0.438894	0.040*	
C16	0.19123 (12)	0.31445 (16)	0.45537 (12)	0.0340 (4)	
H16	0.187115	0.214945	0.434705	0.041*	
C17	0.27739 (11)	0.38811 (17)	0.48284 (12)	0.0333 (3)	
H17	0.332729	0.338914	0.481144	0.040*	
C18	0.28401 (10)	0.53428 (16)	0.51310 (11)	0.0279 (3)	
H18	0.343637	0.584308	0.531424	0.033*	
C19	0.14487 (11)	0.97277 (16)	0.59749 (11)	0.0290 (3)	
H19A	0.133450	1.035458	0.539788	0.035*	0.759 (7)
H19B	0.209846	0.994857	0.644261	0.035*	0.759 (7)
H19C	0.099942	1.029705	0.543008	0.035*	0.241 (7)
H19D	0.210259	1.009976	0.609276	0.035*	0.241 (7)
C20	0.0719 (2)	1.0096 (2)	0.6428 (2)	0.0236 (6)	0.759 (7)
H20	0.006860	0.990264	0.593438	0.028*	0.759 (7)
C21	0.0834 (4)	0.9156 (3)	0.7297 (3)	0.0467 (10)	0.759 (7)
H21A	0.079789	0.811575	0.711351	0.070*	0.759 (7)
H21B	0.032208	0.938305	0.754019	0.070*	0.759 (7)
H21C	0.145684	0.935587	0.780327	0.070*	0.759 (7)
C22	0.0772 (3)	1.1724 (4)	0.6695 (3)	0.0330 (8)	0.759 (7)
H22A	0.141592	1.195593	0.715079	0.049*	0.759 (7)
H22B	0.030125	1.193924	0.699508	0.049*	0.759 (7)
H22C	0.063235	1.232146	0.610940	0.049*	0.759 (7)
C20A	0.1210 (8)	1.0007 (8)	0.6866 (8)	0.040 (2)	0.241 (7)
H20A	0.177371	0.971916	0.744905	0.048*	0.241 (7)
C21A	0.0365 (9)	0.9228 (14)	0.6893 (11)	0.057 (3)	0.241 (7)
H21D	−0.020923	0.961469	0.638568	0.085*	0.241 (7)
H21E	0.031023	0.937251	0.752609	0.085*	0.241 (7)
H21F	0.042711	0.817609	0.678463	0.085*	0.241 (7)
C22A	0.1086 (13)	1.1651 (16)	0.6878 (16)	0.070 (5)	0.241 (7)
H22D	0.138498	1.211442	0.646093	0.106*	0.241 (7)
H22E	0.138969	1.201675	0.754050	0.106*	0.241 (7)
H22F	0.040131	1.188997	0.663855	0.106*	0.241 (7)

### (1Z,2Z)-N1,N2-Diisobutyl-1,2-diphenylethane-1,2-diimine Atomic displacement parameters (Å^2^)

**Table:**

	*U*^11^	*U*^22^	*U*^33^	*U*^12^	*U*^13^	*U*^23^
N1	0.0231 (6)	0.0230 (6)	0.0273 (6)	−0.0019 (4)	0.0135 (5)	−0.0029 (5)
N2	0.0257 (6)	0.0233 (6)	0.0294 (6)	−0.0015 (5)	0.0139 (5)	−0.0021 (5)
C1	0.0207 (6)	0.0175 (6)	0.0288 (7)	−0.0004 (5)	0.0143 (5)	−0.0019 (5)
C2	0.0199 (6)	0.0237 (7)	0.0232 (7)	−0.0014 (5)	0.0094 (5)	0.0024 (5)
C3	0.0251 (7)	0.0174 (6)	0.0289 (7)	0.0012 (5)	0.0161 (6)	−0.0016 (5)
C4	0.0276 (7)	0.0270 (7)	0.0316 (8)	−0.0028 (6)	0.0137 (6)	0.0002 (6)
C5	0.0400 (9)	0.0355 (8)	0.0304 (8)	0.0006 (7)	0.0128 (7)	0.0026 (7)
C6	0.0501 (10)	0.0288 (8)	0.0367 (9)	0.0044 (7)	0.0272 (8)	0.0063 (6)
C7	0.0398 (8)	0.0238 (7)	0.0450 (9)	−0.0030 (6)	0.0291 (7)	0.0009 (6)
C8	0.0269 (7)	0.0229 (7)	0.0347 (8)	−0.0032 (5)	0.0177 (6)	−0.0022 (6)
C9	0.0240 (7)	0.0244 (7)	0.0289 (7)	−0.0025 (5)	0.0121 (6)	0.0001 (6)
C10	0.0225 (7)	0.0292 (7)	0.0280 (7)	−0.0033 (5)	0.0107 (6)	−0.0013 (6)
C11	0.0363 (8)	0.0420 (9)	0.0301 (8)	−0.0055 (7)	0.0096 (7)	0.0023 (7)
C12	0.0347 (8)	0.0293 (8)	0.0335 (8)	−0.0032 (6)	0.0135 (7)	−0.0057 (6)
C13	0.0274 (7)	0.0224 (7)	0.0220 (7)	−0.0037 (5)	0.0134 (5)	−0.0007 (5)
C14	0.0271 (7)	0.0252 (7)	0.0366 (8)	−0.0018 (6)	0.0171 (6)	−0.0007 (6)
C15	0.0343 (8)	0.0275 (8)	0.0436 (9)	−0.0095 (6)	0.0198 (7)	−0.0055 (7)
C16	0.0474 (9)	0.0200 (7)	0.0415 (9)	−0.0024 (6)	0.0242 (8)	−0.0037 (6)
C17	0.0345 (8)	0.0293 (8)	0.0408 (9)	0.0053 (6)	0.0194 (7)	−0.0032 (7)
C18	0.0264 (7)	0.0281 (7)	0.0319 (8)	−0.0019 (6)	0.0137 (6)	−0.0029 (6)
C19	0.0306 (7)	0.0261 (7)	0.0370 (8)	−0.0021 (6)	0.0204 (6)	−0.0048 (6)
C20	0.0219 (13)	0.0252 (10)	0.0252 (13)	0.0030 (8)	0.0105 (10)	−0.0018 (8)
C21	0.092 (3)	0.0257 (12)	0.0375 (17)	−0.0096 (16)	0.0416 (18)	−0.0038 (12)
C22	0.044 (2)	0.0257 (13)	0.0392 (14)	0.0122 (12)	0.0263 (15)	−0.0003 (10)
C20A	0.042 (6)	0.037 (4)	0.043 (5)	0.006 (4)	0.017 (5)	0.001 (3)
C21A	0.065 (7)	0.071 (6)	0.056 (7)	−0.005 (6)	0.048 (6)	−0.016 (6)
C22A	0.062 (10)	0.038 (6)	0.115 (12)	0.010 (6)	0.036 (8)	−0.002 (6)

### (1Z,2Z)-N1,N2-Diisobutyl-1,2-diphenylethane-1,2-diimine Geometric parameters (Å, º)

**Table:**

N1—C1	1.2738 (18)	C14—C15	1.383 (2)
N1—C9	1.4600 (18)	C15—H15	0.9500
N2—C2	1.2694 (17)	C15—C16	1.392 (2)
N2—C19	1.4623 (18)	C16—H16	0.9500
C1—C2	1.5246 (17)	C16—C17	1.378 (2)
C1—C3	1.4915 (19)	C17—H17	0.9500
C2—C13	1.4991 (19)	C17—C18	1.395 (2)
C3—C4	1.392 (2)	C18—H18	0.9500
C3—C8	1.4003 (18)	C19—H19A	0.9900
C4—H4	0.9500	C19—H19B	0.9900
C4—C5	1.394 (2)	C19—H19C	0.9900
C5—H5	0.9500	C19—H19D	0.9900
C5—C6	1.384 (2)	C19—C20	1.512 (2)
C6—H6	0.9500	C19—C20A	1.510 (8)
C6—C7	1.386 (2)	C20—H20	1.0000
C7—H7	0.9500	C20—C21	1.504 (4)
C7—C8	1.387 (2)	C20—C22	1.526 (4)
C8—H8	0.9500	C21—H21A	0.9800
C9—H9A	0.9900	C21—H21B	0.9800
C9—H9B	0.9900	C21—H21C	0.9800
C9—C10	1.530 (2)	C22—H22A	0.9800
C10—H10	1.0000	C22—H22B	0.9800
C10—C11	1.523 (2)	C22—H22C	0.9800
C10—C12	1.520 (2)	C20A—H20A	1.0000
C11—H11A	0.9800	C20A—C21A	1.463 (13)
C11—H11B	0.9800	C20A—C22A	1.507 (14)
C11—H11C	0.9800	C21A—H21D	0.9800
C12—H12A	0.9800	C21A—H21E	0.9800
C12—H12B	0.9800	C21A—H21F	0.9800
C12—H12C	0.9800	C22A—H22D	0.9800
C13—C14	1.4002 (19)	C22A—H22E	0.9800
C13—C18	1.3898 (19)	C22A—H22F	0.9800
C14—H14	0.9500		
			
C1—N1—C9	120.48 (11)	C16—C15—H15	119.9
C2—N2—C19	118.82 (12)	C15—C16—H16	120.2
N1—C1—C2	124.72 (12)	C17—C16—C15	119.63 (14)
N1—C1—C3	119.05 (12)	C17—C16—H16	120.2
C3—C1—C2	116.21 (11)	C16—C17—H17	119.7
N2—C2—C1	124.37 (12)	C16—C17—C18	120.55 (14)
N2—C2—C13	119.40 (12)	C18—C17—H17	119.7
C13—C2—C1	116.21 (11)	C13—C18—C17	120.21 (13)
C4—C3—C1	121.90 (12)	C13—C18—H18	119.9
C4—C3—C8	118.68 (13)	C17—C18—H18	119.9
C8—C3—C1	119.41 (12)	N2—C19—H19A	109.2
C3—C4—H4	119.7	N2—C19—H19B	109.2
C3—C4—C5	120.58 (13)	N2—C19—H19C	108.7
C5—C4—H4	119.7	N2—C19—H19D	108.7
C4—C5—H5	119.9	N2—C19—C20	112.00 (13)
C6—C5—C4	120.22 (15)	N2—C19—C20A	114.3 (3)
C6—C5—H5	119.9	H19A—C19—H19B	107.9
C5—C6—H6	120.2	H19C—C19—H19D	107.6
C5—C6—C7	119.68 (14)	C20—C19—H19A	109.2
C7—C6—H6	120.2	C20—C19—H19B	109.2
C6—C7—H7	119.8	C20A—C19—H19C	108.7
C6—C7—C8	120.39 (14)	C20A—C19—H19D	108.7
C8—C7—H7	119.8	C19—C20—H20	107.8
C3—C8—H8	119.8	C19—C20—C22	110.62 (19)
C7—C8—C3	120.45 (14)	C21—C20—C19	112.2 (3)
C7—C8—H8	119.8	C21—C20—H20	107.8
N1—C9—H9A	109.6	C21—C20—C22	110.5 (2)
N1—C9—H9B	109.6	C22—C20—H20	107.8
N1—C9—C10	110.35 (11)	C20—C21—H21A	109.5
H9A—C9—H9B	108.1	C20—C21—H21B	109.5
C10—C9—H9A	109.6	C20—C21—H21C	109.5
C10—C9—H9B	109.6	H21A—C21—H21B	109.5
C9—C10—H10	108.3	H21A—C21—H21C	109.5
C11—C10—C9	109.30 (12)	H21B—C21—H21C	109.5
C11—C10—H10	108.3	C20—C22—H22A	109.5
C12—C10—C9	111.59 (12)	C20—C22—H22B	109.5
C12—C10—H10	108.3	C20—C22—H22C	109.5
C12—C10—C11	110.82 (13)	H22A—C22—H22B	109.5
C10—C11—H11A	109.5	H22A—C22—H22C	109.5
C10—C11—H11B	109.5	H22B—C22—H22C	109.5
C10—C11—H11C	109.5	C19—C20A—H20A	108.6
H11A—C11—H11B	109.5	C21A—C20A—C19	115.0 (10)
H11A—C11—H11C	109.5	C21A—C20A—H20A	108.6
H11B—C11—H11C	109.5	C21A—C20A—C22A	111.6 (10)
C10—C12—H12A	109.5	C22A—C20A—C19	104.2 (10)
C10—C12—H12B	109.5	C22A—C20A—H20A	108.6
C10—C12—H12C	109.5	C20A—C21A—H21D	109.5
H12A—C12—H12B	109.5	C20A—C21A—H21E	109.5
H12A—C12—H12C	109.5	C20A—C21A—H21F	109.5
H12B—C12—H12C	109.5	H21D—C21A—H21E	109.5
C14—C13—C2	119.60 (12)	H21D—C21A—H21F	109.5
C18—C13—C2	121.48 (12)	H21E—C21A—H21F	109.5
C18—C13—C14	118.91 (13)	C20A—C22A—H22D	109.5
C13—C14—H14	119.7	C20A—C22A—H22E	109.5
C15—C14—C13	120.55 (13)	C20A—C22A—H22F	109.5
C15—C14—H14	119.7	H22D—C22A—H22E	109.5
C14—C15—H15	119.9	H22D—C22A—H22F	109.5
C14—C15—C16	120.15 (14)	H22E—C22A—H22F	109.5
			
N1—C1—C2—N2	88.92 (18)	C2—C13—C14—C15	178.53 (13)
N1—C1—C2—C13	−92.70 (16)	C2—C13—C18—C17	−178.95 (13)
N1—C1—C3—C4	176.57 (13)	C3—C1—C2—N2	−92.90 (16)
N1—C1—C3—C8	−2.69 (18)	C3—C1—C2—C13	85.48 (14)
N1—C9—C10—C11	−176.32 (12)	C3—C4—C5—C6	−0.1 (2)
N1—C9—C10—C12	60.76 (15)	C4—C3—C8—C7	−0.9 (2)
N2—C2—C13—C14	16.3 (2)	C4—C5—C6—C7	−0.4 (2)
N2—C2—C13—C18	−165.14 (13)	C5—C6—C7—C8	0.1 (2)
N2—C19—C20—C21	57.4 (3)	C6—C7—C8—C3	0.5 (2)
N2—C19—C20—C22	−178.7 (2)	C8—C3—C4—C5	0.7 (2)
N2—C19—C20A—C21A	−45.1 (12)	C9—N1—C1—C2	−2.99 (19)
N2—C19—C20A—C22A	−167.6 (7)	C9—N1—C1—C3	178.88 (11)
C1—N1—C9—C10	−148.72 (12)	C13—C14—C15—C16	0.4 (2)
C1—C2—C13—C14	−162.17 (12)	C14—C13—C18—C17	−0.4 (2)
C1—C2—C13—C18	16.39 (19)	C14—C15—C16—C17	−0.3 (2)
C1—C3—C4—C5	−178.58 (13)	C15—C16—C17—C18	−0.1 (2)
C1—C3—C8—C7	178.38 (12)	C16—C17—C18—C13	0.5 (2)
C2—N2—C19—C20	−166.92 (17)	C18—C13—C14—C15	−0.1 (2)
C2—N2—C19—C20A	−134.0 (6)	C19—N2—C2—C1	−0.8 (2)
C2—C1—C3—C4	−1.72 (18)	C19—N2—C2—C13	−179.18 (12)
C2—C1—C3—C8	179.02 (12)		

### (1Z,2Z)-N1,N2-Diisobutyl-1,2-diphenylethane-1,2-diimine Hydrogen-bond geometry (Å, º)

*Cg*1 and *Cg*2 are the centroids of phenyl rings C3–C8 and C13–C18, respectively.

**Table:**

*D*—H···*A*	*D*—H	H···*A*	*D*···*A*	*D*—H···*A*
C6—H6···*Cg*2^i^	0.95	3.23	4.044 (5)	144
C16—H16···*Cg*1^ii^	0.95	2.95	3.76 (7)	144
C21—H21*C*···*Cg*2^iii^	0.98	3.00	3.72 (5)	131

Symmetry codes: (i) *x*, −*y*+1/2, *z*−3/2; (ii) *x*, *y*+1, *z*; (iii) *x*, −*y*+1/2, *z*−1/2.
